# Genomic Instability and Cancer Risk Associated with Erroneous DNA Repair

**DOI:** 10.3390/ijms222212254

**Published:** 2021-11-12

**Authors:** Ken-ichi Yoshioka, Rika Kusumoto-Matsuo, Yusuke Matsuno, Masamichi Ishiai

**Affiliations:** 1 Laboratory of Genome Stability Maintenance, National Cancer Center Research Institute, Tsukiji, Chuo-ku, Tokyo 104-0045, Japan; rikmatsu@ncc.go.jp (R.K.-M.); yumatsun@ncc.go.jp (Y.M.); 2 Department of Applied Chemistry, Faculty of Science, Tokyo University of Science, Kagurazaka, Shinjuku-ku, Tokyo 162-8601, Japan; 3 Central Radioisotope Division, National Cancer Center Research Institute, Tsukiji, Chuo-ku, Tokyo 104-0045, Japan; mishiai@ncc.go.jp

**Keywords:** genomic instability, chromosomal instability (CIN), microsatellite instability (MSI), homologous recombination (HR), non-homologous end-joining (NHEJ), microhomology-mediated end-joining (MMEJ), nucleotide excision repair (NER), mismatch repair (MMR)

## Abstract

Many cancers develop as a consequence of genomic instability, which induces genomic rearrangements and nucleotide mutations. Failure to correct DNA damage in DNA repair defective cells, such as in *BRCA1* and *BRCA2* mutated backgrounds, is directly associated with increased cancer risk. Genomic rearrangement is generally a consequence of erroneous repair of DNA double-strand breaks (DSBs), though paradoxically, many cancers develop in the absence of DNA repair defects. DNA repair systems are essential for cell survival, and in cancers deficient in one repair pathway, other pathways can become upregulated. In this review, we examine the current literature on genomic alterations in cancer cells and the association between these alterations and DNA repair pathway inactivation and upregulation.

## 1. Introduction

Many cancers develop in association with genomic instability, which includes multiple types of genomic alterations, such as nucleotide substitutions and genomic rearrangements [1,2,3]. Recent in vitro studies have suggested a direct association between genomic instability and cancer development, resulting from large-scale mutations followed by clonal evolution of cells abrogated for cancer suppressor genes [4,5]. This instability is unlikely to result from the misincorporation of nucleotides during canonical replication, as these errors are limited, even in a background defective for mismatch repair (MMR), a pathway required to overcome DNA polymerase errors [4]. Therefore, it is purported that clonal evolution of cells abrogated for cancer suppression systems occurs in association with genome destabilization.

**Figure 1:**
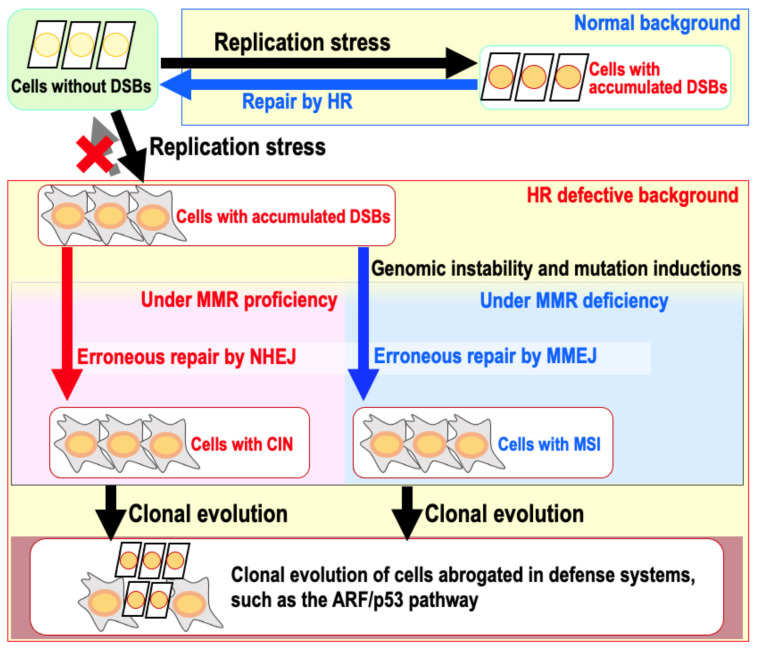
Models for mutagenesis and clonal evolution associated with CIN/MSI induction. Replication stress-associated DSBs are generally repaired by HR under normal conditions. In HR defective backgrounds, these DSBs accumulate and lead to genomic instability through erroneous DSB repair by NHEJ and MMEJ, resulting in CIN and MSI, respectively. Since genomic destabilization is associated with mutagenesis, this further leads to clonal evolution.

Genomic instability is conventionally classified as either chromosomal instability (CIN) or microsatellite instability (MSI) [1]. CIN describes a wide variety of chromosomal abnormalities, including chromosomal rearrangements, deletions, insertions, and amplifications [6,7]. MSI results from the insertion of a few base pairs or deletion mutations induced specifically at repetitive microsatellite loci in MMR-deficient backgrounds [8]. Based on recent studies showing that in mouse embryonic fibroblast cells (MEFs), both CIN and MSI can be induced through the erroneous repair of DSBs arising during replication stress, in which MSI is induced under MMR deficiency [4] (Figure 1). Importantly, genomic destabilization (i.e., induction of genomic instability) results in the clonal evolution of cells mutated in the ARF/p53 pathway. Consistent with this notion, cancer develops through multiple cycles of clonal evolution in association with genomic instability, which results in the extensive accumulation of DSBs [9]. DSBs are toxic DNA lesions that can result in genetic alterations and cell death, such as through apoptosis [10,11,12]. In response to DSBs, ATM and ATR are generally activated to induce cell cycle arrest and DNA repair, or apoptosis if the DSBs are numerous [13,14,15]. DSBs can be repaired by a number of DNA repair pathways, including homologous recombination (HR), non-homologous end-joining (NHEJ), microhomology-mediated end-joining (MMEJ), also known as alternative-NHEJ, and single-strand annealing (SSA). Deficiencies in nucleotide excision repair (NER), a pathway responsible for the repair of DNA helix distorting lesions, including UV photoproducts, is also associated with increased cancer risk [16,17]. DSBs often accumulate even in normal cells when the cells express senescence-associated phenotypes [18,19,20], which are shown in both telomeric and non-telomeric regions. The erroneous repair of these DSBs results in genomic instability.

In this study, we review the current literature on the dysregulation of DNA repair, genome instability, and the cancer risk associated with these events. We also review the risk of genomic destabilization in DNA repair-proficient cell backgrounds, which is associated with senescence-associated phenotypes in response to the accumulation of DSBs. Since these cells can accumulate DSBs in telomeric regions as well as in other regions, we also explore the effects of telomere maintenance in cancer cells, as well as the DNA repair pathways in cancer cells that are induced when multiple different types of DNA damage occur during radiotherapy and chemotherapy.

## 2. HR Deficiency and Increased Cancer Risk

**Figure 2:**
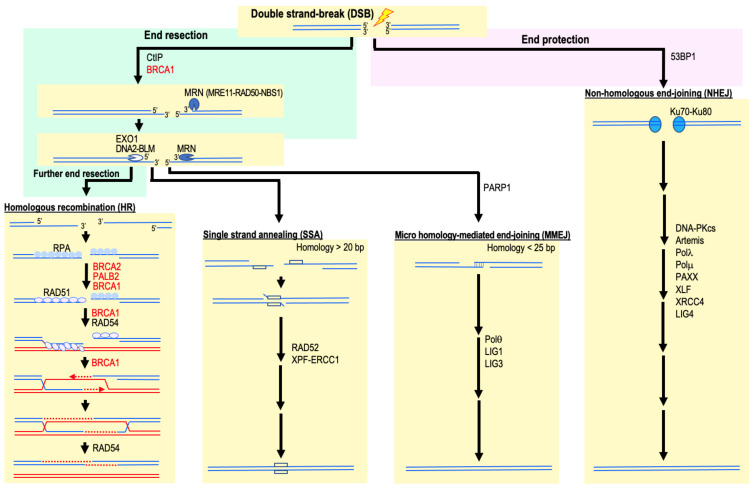
DSB repair pathways. Repair pathway choice is influenced by blocking or initiating end resection. Typical regulatory proteins of these pathways are indicated. At the molecular level, pathway choice is determined by either 53BP1 or BRCA1 and CtIP. CtIP and the MRN complex are involved in extensive 5′ to 3′ resection of the duplex-DNA to generate stretches of single-stranded (ss)-DNA at DNA ends for HR, SSA, and MMEJ. In HR repair, the ssDNA overhangs resulting from DNA end resection are coated with RPA, which is subsequently replaced by RAD51 in a BRCA2-DSS1 complex-dependent manner. RAD51 mediated strand exchange and its association with BRCA1, BRCA2, and RAD54 are essential for the further promotion of the HR pathway.

DSBs are generally repaired by HR, NHEJ, MMEJ, or SSA (Figure 2). At the initial stages of cancer development, cells widely accumulate DSBs in association with replication stress. Prolonged replication stress can lead to the collapse of replication forks and the formation of DSBs. Similarly, DSBs can also be induced when the replication fork encounters unrepaired single-strand DNA breaks (SSBs). These DSBs are repaired predominantly by HR and, if left unrepaired, can lead to genomic destabilization in association with an elevated risk of cancer development [4,21,22,23]. HR relies on the presence of homologous sequences that act as a template for DSB repair. The homologous sequences are frequently homologous chromosomes or, during the repair of replication-associated DSBs, sister chromatids.

DSB repair by HR is initiated by nucleic degradation of the DSB ends, known as DNA end resection (Figure 2). DNA end resection first requires the action of the nucleases Mre11 and CtIP. Mre11 establishes a complex with RAD50 and NBS to form the MRN complex (MRE11–RAD50–NBS1). DNA end resection is regulated by BRCA1 through its interaction with both the MRN complex and CtIP. Subsequently, end resection is completed by Exo1, DNA2, and BLM helicase, leading to the formation of a 3′ single-stranded DNA (ssDNA) tail [24,25]. The resulting ssDNA ends are rapidly coated by RPA protein, making them resistant to further degradation. RPA is then displaced by the recombinase RAD51, which is mediated by BRCA2-DSS1 under the regulation of BRCA1 and PALB2 [24,25]. BRCA1 also stimulates the RAD51-ssDNA nucleoprotein filament to perform a homology search and strand invasion of the filament into homologous duplex DNA, leading to the formation of a displacement loop (D-loop) [24,25]. Mutations in *BRCA1*, *BRCA2*, and *PALB2* are strongly associated with cancer predisposition.

HR is an error-free DNA repair pathway and is required to maintain genome stability; therefore, HR deficiencies are frequently associated with genomic instability and associated cancer predisposition [9]. For example, germline mutations in *BRCA1* and *BRCA2* can increase the risk of developing many cancers with genomic instability, particularly breast and ovarian cancers [26]. Genetic and epigenetic inactivation of other components of the HR machinery has been observed in sporadic cancers, including PALB2, BARD1, RAD51B, RAD51C, and RAD51D [27,28].

## 3. FA Factor Mutation and Increased Cancer Risk

HR is deficient in Fanconi Anemia (FA), a rare genetic disease resulting from a failure in the FA/BRCA DNA repair pathway. To date, 22 FA genes have been identified (*FANCA* to *FANCW*), with mutations in any one of these genes leading to bone marrow failure, developmental abnormalities, and a predisposition to cancer. Cells from FA patients are hypersensitive to DNA inter-strand crosslink (ICL)-inducing agents, such as mitomycin C and cisplatin, and often accumulate chromosomal breaks.

Eight FA gene products (FANCA, FANCB, FANCC, FANCE, FANCF, FANCG, FANCL, and FANCM) assemble into the FA Core Complex, a ubiquitin E3 ligase that monoubiquitinates the FANCD2/FANCI heterodimer (I-D complex) [29,30,31]. Monoubiquitinated I-D complex localizes to sites of DNA damage and interacts with other DNA repair proteins, including other FA factors (FANCD1/BRCA2, FANCJ/BRIP1, FANCN/PALB2, FANCO/RAD51C, FANCR/RAD51, FANCS/BRCA1, FANCU/XRCC2, FANCQ/XPF, FANCP/SLX4, FANCV/REV7, and FANCW/RFWD3), to promote the removal of the ICL, and perform repair via further downstream reactions, including TLS or HR [29,30,31]. FA factors are also responsible for the maintenance of genome stability following replication stress from a variety of sources, including endogenous stress resulting from oncogenes or aldehyde accumulation; DNA damaging agents that disrupt replication, such as hydroxyurea; and low-dose treatment of DNA polymerase inhibitors, such as aphidicolin [32,33,34]. Intriguingly, some FA factors are also required for the maintenance of common fragile sites (CFSs), the fragility of which is thought to be caused by a combination of multiple mechanisms. As recently shown, FANCI and FANCD2 are required for the maintenance of two CFS loci, FRA3B and FRA16D, where the large tumor suppressor genes *FHIT* and *WWOX* reside [32,33,34]. Monoubiquitinated FANCI and FANCD2 accumulate on the CFS loci under mild replication stress conditions. This is associated with the formation of an R-loop consisting of a DNA-RNA hybrid and displaced ssDNA, which constitutes a major threat to genome stability. FANCD2 is required for R-loop resolution [32,33,34]. Collectively, these data reveal a broader role for FA in the maintenance of genome integrity [32,33,34].

Mutations in the FA pathway are associated with predisposition to breast cancer [29,30,35]. Homozygous mutations in *FANCD1/BRCA2* are associated with FA disease, while inherited heterozygous mutations in *FANCD1/BRCA2* are associated with an increased risk of developing breast and ovarian cancers. Similarly, while heterozygous *FANCS/BRCA1* mutations are associated with hereditary breast and ovarian cancer syndromes, biallelic loss of *FANCS/BRCA1* is associated with FA development. Other FA genes, such as *FANCJ/BRIP1* and *FANCN/PALB2*, have also been identified as breast cancer susceptibility genes [29,30,35]. Sporadic alterations in FA genes are frequently observed in many cancers. Over 65% of cancers in public databases have at least one alteration in a FA gene [29,30,36]. While 80% of FA patients have mutations in *FANCA*, *FANCC*, or *FANCG*, somatic FA gene mutations in cancers are distributed evenly. Such differences in mutation distribution in cancer and FA might reflect a functional difference in FA genes in the suppression of FA and cancer [29,30,36]. In addition, copy number alterations in FA genes are often observed in cancer, with many cancers showing upregulation of the expression of FA genes. It is possible that this overexpression relies on coordinated regulation by the Rb/E2F pathway, which contributes to cell proliferation. These findings suggest that many cancers develop with simultaneous mutation and upregulation of FA genes, possibly because some level of DNA repair capacity is beneficial for cancer progression [29,30,36].

## 4. Involvement of NHEJ and MMEJ in Cancer Development

Both CIN and MSI can be induced in a HR-deficient cellular background, which are thought to result from the erroneous repair of DSBs by NHEJ and MMEJ [4,37] (Figure 1). In these contexts, NHEJ and MMEJ contribute to cancer development with progressive genomic destabilization. However, as shown in mouse model studies, cancer development can be promoted by cellular backgrounds mutated in NHEJ and MMEJ factors [38,39,40]. This indicates that cancer is induced by genomic alterations; however, such genomic destabilization can be induced by multiple pathways.

The survival of some cancer cells is dependent on MMEJ. MMEJ is a specialized NHEJ pathway that requires the activity of PARP and DNA Pol θ [41]. *BRCA1*- and *2*-mutated cancers are incredibly sensitive to PARP inhibitors and *Pol θ* knockdown [41,42], suggesting a reliance on MMEJ. Even in cells proficient for HR, PARP inhibitors can significantly sensitize cells to therapeutic agents, such as camptothecin [43], indicating that PARP1-mediated repair pathways contribute to the endurance of DNA damage [44]. Conversely, PARP1 may actively suppress some cancers, since PARP1 knockout mice show increased incidence of liver cancer with advancing age [45]. Thus, the effect of PARP1-mediated DNA repair on cancer suppression and progression may be dependent on the cellular context.

Most CIN-related genomic rearrangement loci are associated with NHEJ in cancer cells [25,46], suggesting that NHEJ is involved in CIN induction and cancer development. Currently, inhibitors of DNA-PK, a mediator of NHEJ, are under clinical trial [47], although a direct association between NHEJ and cancer cell survival remains unclear. Conversely, some cancers may develop as a result of NHEJ deficiency since some cancers develop epigenetic silencing of two genes essential for NHEJ: *KU70* and *KU80* [48,49,50]. The effect of NHEJ on cancer suppression and progression may also be dependent on the cellular context.

## 5. NER Deficiency and Increased Cancer Risk

**Figure 3:**
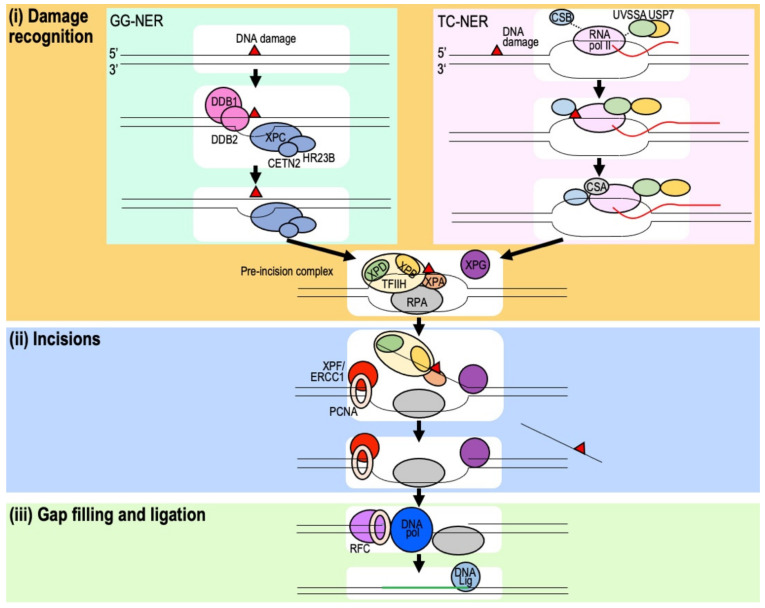
The NER pathway. (**i**) DNA damage can be recognized by XPC/HR23B/CETN2 and/or DDB1/2, which detect helix distortions caused by lesions during GG-NER, or indirectly upon RNA pol II stalling at a DNA lesion during TC-NER. Stalled RNA pol II tightly binds CSB complexed with CSA and UVSSA/USP7. After damage recognition, the TFIIH complex, which includes the helicases XPD and XPB as subunits, is recruited to the site of damage, and TFIIH further recruits XPA and verifies the existence of the damage. RPA binds to ssDNA, and endonuclease XPG is recruited to the complex by TFIIH, forming the TFIIH-XPA-RPA-XPG pre-incision complex. (**ii**) The endonuclease XPF-ERCC1 complex recruited by XPA incises 5′ to the lesion. Subsequent incision by XPG occurs, releasing the oligonucleotide containing the damage. (**iii**) The gap is filled and ligated. Green line: newly synthesized DNA.

NER is required for the removal of DNA adducts that cause helical distortions and can be separated into two main sub-pathways: global genome repair (GG-NER) and transcription-coupled repair (TC-NER) (Figure 3). GG-NER targets helix distorting lesions, UV photolesions, such as (6-4) photoproducts and cyclobutane pyrimidine dimers, and DNA intra-strand crosslinks [51]. By contrast, TC-NER is induced specifically when a lesion blocks the progress of RNA polymerase II (pol II) [52]. These pathways require XPA–G, CSA-B, TTDA, and UV-stimulated scaffold protein A (UVSSA) [16], whose deficiencies are associated with several human autosomal recessive genetic diseases, such as xeroderma pigmentosum (XP), Cockayne syndrome (CS), trichothiodystrophy (TTD), and UV sensitive syndrome (UVSS), respectively [16]. While XP patients have a more than a 1000-fold increased risk of developing cancer [53], CS patients usually do not predispose cancer [54].

The repair pathways for both GG- and TC-NER are similar and share common stages of repair as follows: (i) damage recognition, (ii) DNA incision, and (iii) DNA synthesis and ligation (Figure 3). In GG-NER, helix distortions caused by DNA lesions are recognized by the XPC/HR23B (RAD23B)/CETN2 complex and/or UV-damaged DNA-binding protein 1 (DDB1)/DDB2 (XPE) [55]. By contrast, damage recognition during TC-NER occurs upon RNA pol II stalling at a DNA lesion [51]. During elongation in unperturbed cells, CSB and UVSSA/ubiquitin-specific peptidase 7 (USP7) interacts weakly with RNA pol II but becomes tightly bound to the polymerase, together with CSA, upon RNA pol II stalling [56]. Downstream of damage recognition, GG- and TC-NER follow an identical pathway: the general transcription factor TFIIH, a multimeric complex that includes the subunits XPD, XPB, and TTDA, is recruited to the damage site [56,57]. XPD, a 5′ to 3′ helicase, verifies the existence of the damage along with XPB and XPA, in which XPA is TFIIH-dependently recruited by to chemically altered nucleotides in ssDNA. Replication protein A (RPA), an ssDNA binding protein, is also recruited at this step. XPG recruited to the damage site by TFIIH stabilizes the complex, forming the TFIIH-XPA-RPA-XPG pre-incision complex. This leads to the further recruitment of the structure-specific endonuclease XPF/ERCC1. After the first incision at 5′ to the lesion by XPF/ERCC1, XPG is activated to incise 3′ to the lesion, excising an oligonucleotide of approximately 30 nucleotides containing the lesion. The resulting gap is filled by DNA pol δ, κ, or ε with proliferating cell nuclear antigen (PCNA), replication factor C (RFC) and RPA, and sealed by DNA ligase I or III [58,59].

Genomic rearrangements and mutations are usually induced in skin cancers, and XP patients, deficient in NER, are prone to cancer, including skin cancer; however, it is unclear how NER deficiency is associated with genome destabilization. One of the correlations reported is the involvement of TC-NER, which is through the recombination stimulation at R-loop sites [60]. In fact, recent studies have further revealed that ELOF1 (transcription elongation factor 1), which mediates TC-NER factor assembly by primarily directing RNA polymerase II ubiquitination, suppresses R-loop formation in S phase, which is further associated with the suppression of replication stress and subsequent DSB induction [61,62]. While the role of ELOF1 in TC-NER induction occurs in concert with CSA and CSB, R-loop suppression by ELOF1 is likely independent of CSA- and CSB-dependent functions [61,62].

## 6. Mismatch Repair Deficiency and Increased Cancer Risk

MMR is employed to correct mistakes created by polymerases during replication. Genomic DNA is usually replicated by high fidelity DNA pol δ and ε, and mis-incorporated nucleotides are primarily corrected by the polymerases’ own proofreading systems, with the remaining errors corrected by MMR [17,63,64]. MMR deficiency is associated with Lynch syndrome, also known as hereditary non-polyposis colorectal cancer (HNPCC), and a significantly increased risk of cancers, particularly colorectal, endometrial, stomach, breast, and ovarian cancers [65,66]. These cancers are characterized by MSI and hypermutation and usually have mutations in MMR proteins, particularly subunits of MutSα (MSH2-MSH6 complex) and MutLα (MLH1-PMS2 complex) [65,66]. MMR deficiencies are also observed in sporadic cancers, many of which are caused by epigenetic methylation of *MLH1* [67]. 

MMR proteins function in pathways other than the MMR pathway, including checkpoint activation in response to certain DNA adducts [68] and the suppression of HR between heterologous DNA strands [69]; however, the role of MMR proteins in cancer suppression is still obscure. MMR-dependent checkpoint activation appears to be important for successful chemotherapy with certain DNA damaging agents, such as Temozolomide, which induces O^6^-methylguanine adducts. However, this checkpoint activation does not elicit anticancer effects, as MMR-deficient mice with separation of function mutations, in which the DNA damage checkpoint remains active, are predisposed to MSI-associated cancers [70]. Similarly, MMR-dependent suppression of HR is unlikely to be associated with cancer suppression, as MutSα alone, and not MutLα, is required for effective HR suppression, while both MutSα and MutLα mutations predispose to MSI-positive cancer [69,71,72]. These data imply that the function required for cancer suppression is associated with the canonical function of MMR, i.e., correction of replication errors. Importantly, this type of cancer suppression is tightly associated with the suppression of MSI, since MSI is not observed in normal cells but is induced in MMR-deficient cancer cells [21].

**Figure 4:**
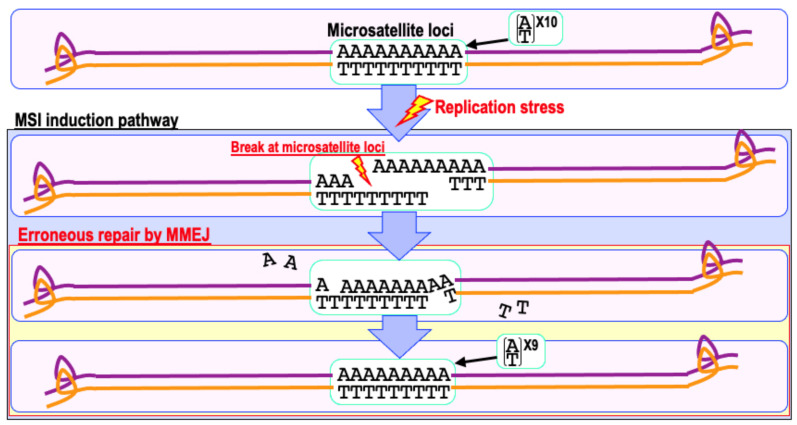
A model of the MSI induction pathway. In an MMR-deficient background, DSBs caused by replication stress at microsatellite loci are effectively repaired by MMEJ. Microsatellite loci can be repeats of a single base or more; thus, erroneous MMEJ can induce multiple types of insertions and deletions of a few bases, i.e., MSI induction, because microhomologous regions can anneal in multiple ways.

Currently, two types of MSI induction pathways are known: (1) a pathway mediated by DNA loop formation [73,74] and (2) a pathway activated by the erroneous repair of replication stress-associated DSBs by MMEJ at microsatellite loci (i.e., patches of short, highly repetitive sequences) [4,37]. The former, originally observed as a pathway induced in trinucleotide repeat disorders [75,76], requires MutSβ (MSH2 and MSH3 complex) for DNA loop formation [73,77,78]. However, given that MSI-positive cancers also present with a MSH2-deficient background, this pathway cannot be the only pathway. Instead, it appears more likely that the latter pathway is the major pathway. In fact, DNA replication stress-associated DSBs are erroneously repaired by MMEJ in MMR deficient cells when these DSBs are not effectively repaired by HR. Since MMEJ requires regions of short homologous repeats to complete DSB repair, microsatellite loci are a natural hotspot for MMEJ activity (Figure 4). However, due to the highly repetitive nature of microsatellites, small insertions or deletions can be induced by MMEJ, resulting in the induction of MSI. Since DSBs are eliminated during this process, CIN-associated genomic alterations are suppressed. In fact, MSI is generally induced as an alternative to CIN in MMR-deficient cancers [1,4,37]. 

Although MMR is required primarily to repair replication errors, the mutation rate associated with canonical replication is still limited even in MMR-deficient cells [5]. A large number of mutations are induced in association with MSI triggered by replication stress-induced DSBs [5]. Indeed, clonal evolution of MMR-deficient MEFs disrupted for the ARF/p53 pathway is induced in association with MSI triggered by replication stress-induced DSBs but is blocked when genome stability is maintained [4,79]. Although this type of clonal evolution is induced even in MMR-proficient MEFs in association with CIN, the associated mutation rate and efficiency of clonal evolution are much higher in MMR-deficient MEFs with MSI. In support of MSI-associated hypermutation induction, low-fidelity TLS polymerases, which lack proofreading activity, are highly induced upon the accumulation of replication stress-induced DSBs [5]. While DNA polymerase δ likely operates in HR-associated DNA synthesis [80,81], TLS are induced single-strand DNA gaps caused under HR deficiency [81,82,83,84,85]. This might provide the major background for the induction of MSI-associated hypermutations.

## 7. Base Excision Repair and Cancer Risk

Base excision repair (BER) is a versatile DNA repair pathway, especially for the repair of non-helix-distorting DNA base lesions, such as those induced by alkylation, oxidation, deamination, and erroneous replication. Many of these lesions are endogenously induced, but some are caused by exogenous chemicals. The major oxidative lesions are 7,8-dihydro-8-oxoguanine (8-oxoguanine) and 5,6-dihydroxy-5,6-dihydrothymine (thymine glycol) [86], while the predominant deleterious lesions induced by alkylating agents, such as methyl methanesulfonate (MMS) and *S*-adenosylmethionine, are 7-methyl guanine and 3-methyl adenine [87]. These lesions are associated with mutations, as incorrect nucleotides are often incorporated opposite these modified bases [88,89]. The BER pathway has been intensively studied and characterized [90].

Associations between inherited BER defects and human genetic disorders have been reported. For example, mutations in the DNA glycosylase *MUTYH* and nth like DNA glycosylase 1 (*NTHL1*), which remove 8-oxoguanine and thymine glycol paired with adenine, respectively, result in colorectal cancer predisposition [91,92,93], while chronic 8-oxoguanine accumulation at telomeres in *OGG1* knockout cells triggers replication stress and significantly increases telomere loss, resulting in chromatin instability [94]. However, the contribution of BER defects to genomic instability and associated cancer is likely to be less than those of other major repair-pathway defects.

## 8. Telomere Maintenance and Cancer

Telomeres are repetitive TTAGGG sequences at the ends of chromosomes. The 3′ end of such sequences are over-hanging, enabling the formation of T-loops coordinated by the shelterin complex (comprising TRF1, TRF2, POT1, TPP1, TIN2, and RAP1) [95,96]. The chromosome ends are widely protected by T-loop formation from the degradation and the initiation of erroneous DSB repair [95,96]. In stem cells, telomere length is maintained by telomerase, which is recruited to telomeric regions by the shelterin complex [95,96]. Maintenance of telomere length is usually dependent on the expression of TERT, a subunit of telomerase. Unlike stem cells, the gene coding TERT in differentiated cells is epigenetically silenced [95,96]; therefore, replication leads to telomere shortening and cellular senescence. Shortened telomeres are detected as DSBs, which results in ATM and ATR activation and aberrant genomic rearrangements [95,96]. These catastrophic events are termed telomere crisis. Thus, many cancer stem cells rely on TERT for telomere maintenance [97,98]. Alternatively, other cancers, such as a type of glioblastoma, rely on HR for the synthesis of telomeres [95,99,100,101]. Thus, while TERT appears to be non-essential, the maintenance of telomere length is likely to be required for cancer development. In most cases, telomere-maintenance activity is likely acquired in association with the development of cancer stem cells [95,97,102,103].

## 9. The Risk of Genomic Destabilization in a Repair-Proficient Background

Cancer usually develops as a result of genomic instability; however, cancers that develop within the context of a background of hereditary mutations in DNA repair pathways are infrequent [104,105,106,107,108]. How cells proficient for DNA repair become subject to genomic destabilization is unclear; however, it is likely that cancer development in these cells is associated with cells entering a state of senescence [4,37]. Senescent cells, and cells in aging organs, generally accumulate DSBs [18]. In addition, senescence-associated phenotypes are induced in response to DSBs that risk genome stability and the accumulation of cytosolic DNA arising from genome instability, especially CIN [109,110,111]. Thus, the senescent phenotype is generally associated with increased genome instability. Consistent with such an argument, cancer rates, especially those associated with genomic instability, increase in association with age [112,113]. Indeed, BLOOM, a syndrome associated with premature signs of aging, is associated with different types of cancer predisposition, in which genomic instability plays an important part [114,115]. 

The question is, how do normal cells with a senescence-associated phenotype become defective in DSB repair? One possibility is the alteration of chromosome states. In fact, H2AX, which is required for efficient damage responses and repair, is largely downregulated when the growth rate of normal cells slows down [116]. These cells can still repair DSBs directly induced by γ-ray irradiation because H2AX is transiently upregulated in response to these DSBs, which is dependent on ATM and SIRT6 [117]. However, cells in such a state are specifically defective in the repair of replication stress-associated DSBs [4,37]. Therefore, persistent DSBs accumulate following oncogene acceleration, growth stimulation, and exogenous stresses, such as γ-ray irradiation. This results in the accumulation of persistent DSBs in the following S phase, associated with increased replication stress [5,109,118]. Replication stress-associated DSBs accumulate in cells at an initial stage of cancer development, supporting the hypothesis that the accumulation of replication stress-associated DSBs increases the risk of cancer development [5,109,118].

## 10. Repair Pathways Activated by Chemo- and Radiotherapy-Induced DNA Damage

DNA damaging agents have been employed as cancer therapeutics for many years, as the accumulation of DNA damage often leads to cell death. This damage can be in the form of DSBs, induced by radiation therapy or drugs, such as doxorubicin and camptothecin, DNA adducts, such as DNA crosslinks induced by cisplatin, methylation damage caused by Temozolomide, or the incorporation of pyrimidine analogs, such as 5-fluorouracil (5-FU), during replication. Cell death is achieved primarily through apoptosis; however, resistance to such treatments can be acquired via a number of different mechanisms, including stress-induced mutagenesis [119,120]. For camptothecin treatment, resistance likely occurs through genomic destabilization associated with replication stress-induced DSBs. While these DSBs induce apoptosis in many cancer cells, a minority of cells survive treatment, with the erroneous repair of camptothecin-associated DSBs leading to genomic rearrangements and destabilization, further risking clonal evolution of camptothecin-resistance cells [4]. Replication stress-associated DSBs similarly accumulate following treatment with ionizing radiation: while DSBs induced directly by radiation are usually repairable, persistent DSBs can be introduced during the following S phase in association with replication stress [5,117,118].

Temozolomide resistance is thought to be attributable to epigenetic silencing of the *O^6^-methylguanine-DNA methyltransferase* (*MGMT*) gene [121]. The toxic effect of Temozolomide is primarily caused by O^6^-methylguanine adducts, which are recognized in a MMR-dependent manner and activate both ATM and ATR kinases, leading to apoptosis and cell death [122]. However, MGMT is able to selectively remove these adducts; thus, cancers expressing little or no MGMT, such as MGMT negative glioblastomas, remain sensitive to treatment [123]. Although chemotherapy often results in therapeutic resistance, the mechanism by which cells acquire resistance to Temozolomide is unclear.

## 11. Perspectives

Deficiencies in many DNA-repair pathways are associated with genomic instability and an increased risk of cancer. The risk of genomic instability can also be increased even in cells not mutated in repair systems when cells express senescence-associated phenotypes, such as those induced by aberrant growth stimulation, oncogene acceleration, and exogenous stresses, such as radiation exposure. Since most human cancers develop in the absence of hereditary mutations in DNA-repair systems but still exhibit genomic instability, most cancers may be a consequence of a cellular state that favors genomic destabilization.

CIN-associated genomic alterations are triggered by replication stress-associated DSBs and are induced largely by erroneous NHEJ repair, particularly when those DSBs are not effectively repaired by HR. This argument is largely supported by the carryover of replication stress-associated DSBs into M phase, which causes chromosomal mis-segregation and tetraploidy in the following G1 phase [124]. By contrast, MSI is induced in MMR-deficient cells by replication stress-associated DSBs and erroneous MMEJ repair at microsatellite loci, during which CIN induction is suppressed. Given that most major cancers develop as a consequence of genomic instability, the accumulation of replication stress-associated DSBs is the major risk factor for the development of cancer, in which cells usually express senescence-associated phenotypes.

Most cancers are inevitably induced by genomic instability. Based on recent reports, it is likely that many of these cancers are caused by genomic destabilization triggered by replication stress-associated DSBs. However, questions remain about how normal cells become defective in repairing replication stress-associated DSBs and why these DSBs are not effectively repaired. Importantly, these recent reports also raise an attractive hypothesis, i.e., many of those cancers could be avoided if genome stability could be maintained. Thus, a future direction to study should be to determine whether or not cancers are avoidable. To address this, it will be important to understand how genome stability can be continuously maintained and how DSBs that accumulate in cellular states that favor genomic destabilization are repaired. 

## References

[1] Lengauer C., Kinzler K.W., Vogelstein B. (1997). Genetic Instability in Colorectal Cancers. Nature.

[2] Lengauer C., Kinzler K.W., Vogelstein B. (1998). Genetic Instabilities in Human Cancers. Nature.

[3] Negrini S., Gorgoulis V.G., Halazonetis T.D. (2010). Genomic Instability—An Evolving Hallmark of Cancer. Nat. Rev. Mol. Cell Biol..

[4] Matsuno Y., Atsumi Y., Shimizu A., Katayama K., Fujimori H., Hyodo M., Minakawa Y., Nakatsu Y., Kaneko S., Hamamoto R. (2019). Replication Stress Triggers Microsatellite Destabilization and Hypermutation Leading to Clonal Expansion in Vitro. Nat. Commun..

[5] Matsuno Y., Hyodo M., Suzuki M., Tanaka Y., Horikoshi Y., Murakami Y., Torigoe H., Mano H., Tashiro S., Yoshioka K. (2021). Replication-Stress-Associated DSBs Induced by Ionizing Radiation Risk Genomic Destabilization and Associated Clonal Evolution. iScience.

[6] Rajagopalan H., Lengauer C. (2004). Aneuploidy and Cancer. Nature.

[7] Korbel J.O., Campbell P.J. (2013). Criteria for Inference of Chromothripsis in Cancer Genomes. Cell.

[8] Woerner S.M., Kloor M., von Knebel Doeberitz M., Gebert J.F. (2006). Microsatellite Instability in the Development of DNA Mismatch Repair Deficient Tumors. Cancer Biomark..

[9] Bishop A.J.R., Schiestl R.H. (2002). Homologous Recombination and Its Role in Carcinogenesis. J. Biomed. Biotechnol..

[10] Hoeijmakers J.H.J. (2009). DNA Damage, Aging, and Cancer. N. Engl. J. Med..

[11] Ciccia A., Elledge S.J. (2010). The DNA Damage Response: Making It Safe to Play with Knives. Mol. Cell.

[12] Sebastian R., Raghavan S.C. (2016). Induction of DNA Damage and Erroneous Repair Can Explain Genomic Instability Caused by Endosulfan. Carcinogenesis.

[13] Abraham R.T. (2001). Cell Cycle Checkpoint Signaling through the ATM and ATR Kinases. Genes Dev..

[14] Maréchal A., Zou L. (2013). DNA Damage Sensing by the ATM and ATR Kinases. Cold Spring Harb. Perspect. Biol..

[15] Blackford A.N., Jackson S.P. (2017). ATM, ATR, and DNA-PK: The Trinity at the Heart of the DNA Damage Response. Mol. Cell.

[16] Marteijn J.A., Lans H., Vermeulen W., Hoeijmakers J.H.J. (2014). Understanding Nucleotide Excision Repair and Its Roles in Cancer and Ageing. Nat. Rev. Mol. Cell Biol..

[17] Modrich P., Lahue R. (1996). Mismatch Repair in Replication Fidelity, Genetic Recombination, and Cancer Biology. Annu. Rev. Biochem..

[18] Sedelnikova O.A., Horikawa I., Zimonjic D.B., Popescu N.C., Bonner W.M., Barrett J.C. (2004). Senescing Human Cells and Ageing Mice Accumulate DNA Lesions with Unrepairable Double-Strand Breaks. Nat. Cell Biol..

[19] Rodier F., Coppé J.-P., Patil C.K., Hoeijmakers W.A.M., Muñoz D.P., Raza S.R., Freund A., Campeau E., Davalos A.R., Campisi J. (2009). Persistent DNA Damage Signaling Triggers Senescence-Associated Inflammatory Cytokine Secretion. Nat. Cell Biol..

[20] Fumagalli M., Rossiello F., Clerici M., Barozzi S., Cittaro D., Kaplunov J.M., Bucci G., Dobreva M., Matti V., Beausejour C.M. (2012). Telomeric DNA Damage Is Irreparable and Causes Persistent DNA-Damage-Response Activation. Nat. Cell Biol..

[21] Fujimori H., Hyodo M., Matsuno Y., Shimizu A., Minakawa Y., Atsumi Y., Nakatsu Y., Tsuzuki T., Murakami Y., Yoshioka K. (2019). Mismatch Repair Dependence of Replication Stress-Associated DSB Recognition and Repair. Heliyon.

[22] Petermann E., Orta M.L., Issaeva N., Schultz N., Helleday T. (2010). Hydroxyurea-Stalled Replication Forks Become Progressively Inactivated and Require Two Different RAD51-Mediated Pathways for Restart and Repair. Mol. Cell.

[23] Tsvetkova A., Ozerov I.V., Pustovalova M., Grekhova A., Eremin P., Vorobyeva N., Eremin I., Pulin A., Zorin V., Kopnin P. (2017). ΓH2AX, 53BP1 and Rad51 Protein Foci Changes in Mesenchymal Stem Cells during Prolonged X-Ray Irradiation. Oncotarget.

[24] Tarsounas M., Sung P. (2020). The Antitumorigenic Roles of BRCA1–BARD1 in DNA Repair and Replication. Nat. Rev. Mol. Cell Biol..

[25] Scully R., Panday A., Elango R., Willis N.A. (2019). DNA Double-Strand Break Repair-Pathway Choice in Somatic Mammalian Cells. Nat. Rev. Mol. Cell Biol..

[26] Bayraktar S., Arun B. (2017). BRCA Mutation Genetic Testing Implications in the United States. Breast.

[27] Bell D., Berchuck A., Birrer M., Chien J., Cramer D.W., Dao F., Dhir R., Disaia P., Gabra H., Glenn P. (2011). Integrated Genomic Analyses of Ovarian Carcinoma. Nature.

[28] Riaz N., Blecua P., Lim R.S., Shen R., Higginson D.S., Weinhold N., Norton L., Weigelt B., Powell S.N., Reis-Filho J.S. (2017). Pan-Cancer Analysis of Bi-Allelic Alterations in Homologous Recombination DNA Repair Genes. Nat. Commun..

[29] Niraj J., Färkkilä A., D’Andrea A.D. (2019). The Fanconi Anemia Pathway in Cancer. Annu. Rev. Cancer Biol..

[30] Nalepa G., Clapp D.W. (2018). Fanconi Anaemia and Cancer: An Intricate Relationship. Nat. Rev. Cancer.

[31] Ishiai M., Sato K., Tomida J., Kitao H., Kurumizaka H., Takata M. (2017). Activation of the FA Pathway Mediated by Phosphorylation and Ubiquitination. Mutat. Res.-Fundam. Mol. Mech. Mutagenesis.

[32] Madireddy A., Kosiyatrakul S.T., Boisvert R.A., Herrera-Moyano E., García-Rubio M.L., Gerhardt J., Vuono E.A., Owen N., Yan Z., Olson S. (2016). FANCD2 Facilitates Replication through Common Fragile Sites. Mol. Cell.

[33] Okamoto Y., Iwasaki W.M., Kugou K., Takahashi K.K., Oda A., Sato K., Kobayashi W., Kawai H., Sakasai R., Takaori-Kondo A. (2018). Replication Stress Induces Accumulation of FANCD2 at Central Region of Large Fragile Genes. Nucleic Acids Res..

[34] Okamoto Y., Hejna J., Takata M. (2019). Regulation of R-Loops and Genome Instability in Fanconi Anemia. J. Biochem..

[35] Fang C.B., Wu H.T., Zhang M.L., Liu J., Zhang G.J. (2020). Fanconi Anemia Pathway: Mechanisms of Breast Cancer Predisposition Development and Potential Therapeutic Targets. Front. Cell Dev. Biol..

[36] Liu W., Palovcak A., Li F., Zafar A., Yuan F., Zhang Y. (2020). Fanconi Anemia Pathway as a Prospective Target for Cancer Intervention. Cell Biosci..

[37] Yoshioka K., Matsuno Y. (2020). Genomic Destabilization and Its Associated Mutagenesis Increase with Senescence-associated Phenotype Expression. Cancer Sci..

[38] Difilippantonio M.J., Zhu J., Chen H.T., Meffre E., Nussenzweig M.C., Max E.E., Ried T., Nussenzweig A. (2000). DNA Repair Protein Ku80 Suppresses Chromosomal Aberrations and Malignant Transformation. Nature.

[39] Espejel S., Martín M., Klatt P., Martín-Caballero J., Flores J.M., Blasco M.A. (2004). Shorter Telomeres, Accelerated Ageing and Increased Lymphoma in DNA-PKcs-deficient Mice. EMBO Rep..

[40] Wyatt D.W., Feng W., Conlin M.P., Yousefzadeh M.J., Roberts S.A., Mieczkowski P., Wood R.D., Gupta G.P., Ramsden D.A. (2016). Essential Roles for Polymerase θ-Mediated End Joining in the Repair of Chromosome Breaks. Mol. Cell.

[41] Patterson-Fortin J., D’Andrea A.D. (2020). Exploiting the Microhomology-Mediated End-Joining Pathway in Cancer Therapy. Cancer Res..

[42] Farmer H., McCabe N., Lord C.J., Tutt A.N.J., Johnson D.A., Richardson T.B., Santarosa M., Dillon K.J., Hickson I., Knights C. (2005). Targeting the DNA Repair Defect in BRCA Mutant Cells as a Therapeutic Strategy. Nature.

[43] Atsumi Y., Inase A., Osawa T., Sugihara E., Sakasai R., Fujimori H., Teraoka H., Saya H., Kanno M., Tashiro F. (2013). The Arf/P53 Protein Module, Which Induces Apoptosis, Down-Regulates Histone H2AX to Allow Normal Cells to Survive in the Presence of Anti-Cancer Drugs. J. Biol. Chem..

[44] Matsuno Y., Hyodo M., Fujimori H., Shimizu A., Yoshioka K. (2018). Sensitization of Cancer Cells to Radiation and Topoisomerase I Inhibitor Camptothecin Using Inhibitors of PARP and Other Signaling Molecules. Cancers.

[45] Nozaki T., Fujihara H., Watanabe M., Tsutsumi M., Nakamoto K., Kusuoka O., Kamada N., Suzuki H., Nakagama H., Sugimura T. (2003). Parp-1 Deficiency Implicated in Colon and Liver Tumorigenesis Induced by Azoxymethane. Cancer Sci..

[46] Wilson T.E., Sunder S. (2020). Double-Strand Breaks in Motion: Implications for Chromosomal Rearrangement. Curr. Genet..

[47] Van Bussel M.T.J., Awada A., de Jonge M.J.A., Mau-Sørensen M., Nielsen D., Schöffski P., Verheul H.M.W., Sarholz B., Berghoff K., el Bawab S. (2021). A First-in-Man Phase 1 Study of the DNA-Dependent Protein Kinase Inhibitor Peposertib (Formerly M3814) in Patients with Advanced Solid Tumours. Br. J. Cancer.

[48] Lee M.N., Tseng R.C., Hsu H.S., Chen J.Y., Tzao C., Ho W.L., Wang Y.C. (2007). Epigenetic Inactivation of the Chromosomal Stability Control Genes BRCA1 BRCA2, and XRCC5 in Non-Small Cell Lung Cancer. Clin. Cancer Res..

[49] Tryndyak V.P., Kovalchuk O., Muskhelishvili L., Montgomery B., Rodrigues-Juarez R., Melnyk S., Ross S.A., Beland F.A., Pogribny I.P. (2007). Epigenetic Reprogramming of Liver Cells in Tamoxifen-Induced Rat Hepatocarcinogenesis. Mol. Carcinog..

[50] Zhou C., Tang H., Yu J., Zhuang D., Zhang H. (2015). Blood-Based DNA Methylation of DNA Repair Genes in the Non-Homologous End-Joining (NEHJ) Pathway in Patient with Glioma. Int. J. Clin. Exp. Pathol..

[51] Friedberg E.C., Walker G.C., Siede W., Wood R.D., Schultz R.A., Ellenberger T. (2005). DNA Repair and Mutagenesis.

[52] Hu J., Selby C.P., Adar S., Adebali O., Sancar A. (2017). Molecular Mechanisms and Genomic Maps of DNA Excision Repair in *Escherichia Coli* and Humans. J. Biol. Chem..

[53] Lehmann J., Seebode C., Martens M.C., Emmert S. (2018). Xeroderma Pigmentosum—Facts and Perspectives. Anticancer Res..

[54] Reid-Bayliss K.S., Arron S.T., Loeb L.A., Bezrookove V., Cleaver J.E. (2016). Why Cockayne Syndrome Patients Do Not Get Cancer despite Their DNA Repair Deficiency. Proc. Natl. Acad. Sci. USA.

[55] Sugasawa K. (2019). Mechanism and regulation of DNA damage recognition in mammalian nucleotide excision repair. Enzymes.

[56] Van der Weegen Y., Golan-Berman H., Mevissen T.E.T., Apelt K., González-Prieto R., Goedhart J., Heilbrun E.E., Vertegaal A.C.O., van den Heuvel D., Walter J.C. (2020). The Cooperative Action of CSB, CSA, and UVSSA Target TFIIH to DNA Damage-Stalled RNA Polymerase II. Nat. Commun..

[57] Oksenych V., de Jesus B.B., Zhovmer A., Egly J.M., Coin F. (2009). Molecular Insights into the Recruitment of TFIIH to Sites of DNA Damage. EMBO J..

[58] Ogi T., Limsirichaikul S., Overmeer R.M., Volker M., Takenaka K., Cloney R., Nakazawa Y., Niimi A., Miki Y., Jaspers N.G. (2010). Three DNA Polymerases, Recruited by Different Mechanisms, Carry Out NER Repair Synthesis in Human Cells. Mol. Cell.

[59] Moser J., Kool H., Giakzidis I., Caldecott K., Mullenders L.H.F., Fousteri M.I. (2007). Sealing of Chromosomal DNA Nicks during Nucleotide Excision Repair Requires XRCC1 and DNA Ligase IIIα in a Cell-Cycle-Specific Manner. Mol. Cell.

[60] Sollier J., Stork C.T., García-Rubio M.L., Paulsen R.D., Aguilera A., Cimprich K.A. (2014). Transcription-Coupled Nucleotide Excision Repair Factors Promote R-Loop-Induced Genome Instability. Mol. Cell.

[61] Geijer M.E., Zhou D., Selvam K., Steurer B., Mukherjee C., Evers B., Cugusi S., van Toorn M., van der Woude M., Janssens R.C. (2021). Elongation Factor ELOF1 Drives Transcription-Coupled Repair and Prevents Genome Instability. Nat. Cell Biol..

[62] Van der Weegen Y., de Lint K., van den Heuvel D., Nakazawa Y., Mevissen T.E.T., van Schie J.J.M., San Martin Alonso M., Boer D.E.C., González-Prieto R., Narayanan I.V. (2021). ELOF1 Is a Transcription-Coupled DNA Repair Factor That Directs RNA Polymerase II Ubiquitylation. Nat. Cell Biol..

[63] Burgers P.M.J., Kunkel T.A. (2017). Eukaryotic DNA Replication Fork. Annu. Rev. Biochem..

[64] Cortez D. (2019). Replication-Coupled DNA Repair. Mol. Cell.

[65] Baretti M., Le D.T. (2018). DNA Mismatch Repair in Cancer. Pharmacol. Ther..

[66] Biller L.H., Syngal S., Yurgelun M.B. (2019). Recent Advances in Lynch Syndrome. Fam. Cancer.

[67] Gao A., Guo M. (2020). Epigenetic Based Synthetic Lethal Strategies in Human Cancers. Biomark. Res..

[68] Gupta D., Heinen C.D. (2019). The Mismatch Repair-Dependent DNA Damage Response: Mechanisms and Implications. DNA Repair.

[69] Anand R., Beach A., Li K., Haber J. (2017). Rad51-Mediated Double-Strand Break Repair and Mismatch Correction of Divergent Substrates. Nature.

[70] Thomas A., Tanaka M., Trepel J., Reinhold W.C., Rajapakse V.N., Pommier Y. (2017). Temozolomide in the Era of Precision Medicine. Cancer Res..

[71] Cortes-Ciriano I., Lee S., Park W.-Y., Kim T.-M., Park P.J. (2017). A Molecular Portrait of Microsatellite Instability across Multiple Cancers. Nat. Commun..

[72] Cortés-Ciriano I., Lee J.J.K., Xi R., Jain D., Jung Y.L., Yang L., Gordenin D., Klimczak L.J., Zhang C.Z., Pellman D.S. (2020). Comprehensive Analysis of Chromothripsis in 2,658 Human Cancers Using Whole-Genome Sequencing. Nat. Genet..

[73] Palombo F., Iaccarino I., Nakajima E., Ikejima M., Shimada T., Jiricny J. (1996). HMutSβ, a Heterodimer of HMSH2 and HMSH3, Binds to Insertion/Deletion Loops in DNA. Curr. Biol..

[74] Li G.-M. (2007). Mechanisms and Functions of DNA Mismatch Repair. Cell Res..

[75] Gannon A.-M.M., Frizzell A., Healy E., Lahue R.S. (2012). MutSβ and Histone Deacetylase Complexes Promote Expansions of Trinucleotide Repeats in Human Cells. Nucleic Acids Res..

[76] Williams G.M., Surtees J.A. (2015). MSH3 Promotes Dynamic Behavior of Trinucleotide Repeat Tracts In Vivo. Genetics.

[77] Allen D.J., Makhov A., Grilley M., Taylor J., Thresher R., Modrich P., Griffith J. (1997). MutS Mediates Heteroduplex Loop Formation by a Translocation Mechanism. EMBO J..

[78] Habraken Y., Sung P., Prakash L., Prakash S. (1996). Binding of Insertion/Deletion DNA Mismatches by the Heterodimer of Yeast Mismatch Repair Proteins MSH2 and MSH3. Curr. Biol..

[79] Matsuno Y., Atsumi Y., Alauddin M.D., Rana M.D.M., Fujimori H., Hyodo M., Shimizu A., Ikuta T., Tani H., Torigoe H. (2020). Resveratrol and Its Related Polyphenols Contribute to the Maintenance of Genome Stability. Sci. Rep..

[80] McVey M., Khodaverdian V.Y., Meyer D., Cerqueira P.G., Heyer W.-D. (2016). Eukaryotic DNA Polymerases in Homologous Recombination. Annu. Rev. Genet..

[81] Conde C.D., Petronczki Ö.Y., Baris S., Willmann K.L., Girardi E., Salzer E., Weitzer S., Ardy R.C., Krolo A., Ijspeert H. (2019). Polymerase δ Deficiency Causes Syndromic Immunodeficiency with Replicative Stress. J. Clin. Investig..

[82] Wong R.P., García-Rodríguez N., Zilio N., Hanulová M., Ulrich H.D. (2020). Processing of DNA Polymerase-Blocking Lesions during Genome Replication Is Spatially and Temporally Segregated from Replication Forks. Mol. Cell.

[83] Ma X., Tang T.-S., Guo C. (2020). Regulation of Translesion DNA Synthesis in Mammalian Cells. Environ. Mol. Mutagenesis.

[84] Nayak S., Calvo J.A., Cong K., Peng M., Berthiaume E., Jackson J., Zaino A.M., Vindigni A., Hadden M.K., Cantor S.B. (2020). Inhibition of the Translesion Synthesis Polymerase REV1 Exploits Replication Gaps as a Cancer Vulnerability. Sci. Adv..

[85] Taglialatela A., Leuzzi G., Sannino V., Cuella-Martin R., Huang J.-W., Wu-Baer F., Baer R., Costanzo V., Ciccia A. (2021). REV1-Polζ Maintains the Viability of Homologous Recombination-Deficient Cancer Cells through Mutagenic Repair of PRIMPOL-Dependent SsDNA Gaps. Mol. Cell.

[86] Marnett L.J. (2000). Oxyradicals and DNA Damage. Carcinogenesis.

[87] Sedgwick B., Bates P.A., Paik J., Jacobs S.C., Lindahl T. (2007). Repair of Alkylated DNA: Recent Advances. DNA Repair.

[88] Cooke M.S., Evans M.D., Dizdaroglu M., Lunec J. (2003). Oxidative DNA Damage: Mechanisms, Mutation, and Disease. FASEB J..

[89] Fu D., Calvo J.A., Samson L.D. (2012). Balancing Repair and Tolerance of DNA Damage Caused by Alkylating Agents. Nat. Rev. Cancer.

[90] Lee T.H., Kang T.H. (2019). DNA Oxidation and Excision Repair Pathways. Int. J. Mol. Sci..

[91] Chow E., Thirlwell C., Macrae F., Lipton L. (2004). Colorectal Cancer and Inherited Mutations in Base-Excision Repair. Lancet Oncol..

[92] Das L., Quintana V.G., Sweasy J.B. (2020). NTHL1 in Genomic Integrity, Aging and Cancer. DNA Repair.

[93] Weren R.D.A., Ligtenberg M.J.L., Geurts van Kessel A., de Voer R.M., Hoogerbrugge N., Kuiper R.P. (2018). NTHL1 and MUTYH Polyposis Syndromes: Two Sides of the Same Coin?. J. Pathol..

[94] Fouquerel E., Barnes R.P., Uttam S., Watkins S.C., Bruchez M.P., Opresko P.L. (2019). Targeted and Persistent 8-Oxoguanine Base Damage at Telomeres Promotes Telomere Loss and Crisis. Mol. Cell.

[95] Maciejowski J., de Lange T. (2017). Telomeres in Cancer: Tumour Suppression and Genome Instability. Nat. Rev. Mol. Cell Biol..

[96] Bernal A., Tusell L. (2018). Telomeres: Implications for Cancer Development. Int. J. Mol. Sci..

[97] Hannen R., Bartsch J.W. (2018). Essential Roles of Telomerase Reverse Transcriptase HTERT in Cancer Stemness and Metastasis. FEBS Lett..

[98] Campbell P.J., Getz G., Korbel J.O., Stuart J.M., Jennings J.L., Stein L.D., Perry M.D., Nahal-Bose H.K., Ouellette B.F.F., Li C.H.C. (2020). Pan-Cancer Analysis of Whole Genomes. Nature.

[99] Diplas B.H., He X., Brosnan-Cashman J.A., Liu H., Chen L.H., Wang Z., Moure C.J., Killela P.J., Loriaux D.B., Lipp E.S. (2018). The Genomic Landscape of TERT Promoter Wildtype-IDH Wildtype Glioblastoma. Nat. Commun..

[100] Williams E.A., Miller J.J., Tummala S.S., Penson T., Iafrate A.J., Juratli T.A., Cahill D.P. (2018). TERT Promoter Wild-Type Glioblastomas Show Distinct Clinical Features and Frequent PI3K Pathway Mutations. Acta Neuropathol. Commun..

[101] Mao P., Liu J., Zhang Z., Zhang H., Liu H., Gao S., Rong Y.S., Zhao Y. (2016). Homologous Recombination-Dependent Repair of Telomeric DSBs in Proliferating Human Cells. Nat. Commun..

[102] Keefe D.L. (2020). Telomeres and Genomic Instability during Early Development. Eur. J. Med. Genet..

[103] Noureen N., Wu S., Lv Y., Yang J., Alfred Yung W.K., Gelfond J., Wang X., Koul D., Ludlow A., Zheng S. (2021). Integrated Analysis of Telomerase Enzymatic Activity Unravels an Association with Cancer Stemness and Proliferation. Nat. Commun..

[104] Stephens P.J., McBride D.J., Lin M.-L., Varela I., Pleasance E.D., Simpson J.T., Stebbings L.A., Leroy C., Edkins S., Mudie L.J. (2009). Complex Landscapes of Somatic Rearrangement in Human Breast Cancer Genomes. Nature.

[105] Pleasance E.D., Cheetham R.K., Stephens P.J., McBride D.J., Humphray S.J., Greenman C.D., Varela I., Lin M.-L., Ordóñez G.R., Bignell G.R. (2010). A Comprehensive Catalogue of Somatic Mutations from a Human Cancer Genome. Nature.

[106] Tomasetti C., Li L., Vogelstein B. (2017). Stem Cell Divisions, Somatic Mutations, Cancer Etiology, and Cancer Prevention. Science.

[107] Alexandrov L.B., Nik-Zainal S., Wedge D.C., Aparicio S.A.J.R., Behjati S., Biankin A.V., Bignell G.R., Bolli N., Borg A., Børresen-Dale A.L. (2013). Signatures of Mutational Processes in Human Cancer. Nature.

[108] Alexandrov L.B., Kim J., Haradhvala N.J., Huang M.N., Tian Ng A.W., Wu Y., Boot A., Covington K.R., Gordenin D.A., Bergstrom E.N. (2020). The Repertoire of Mutational Signatures in Human Cancer. Nature.

[109] Gorgoulis V.G., Vassiliou L.-V.V.F., Karakaidos P., Zacharatos P., Kotsinas A., Liloglou T.T., Venere M., DiTullio R.A., Kastrinakis N.G., Levy B. (2005). Activation of the DNA Damage Checkpoint and Genomic Instability in Human Precancerous Lesions. Nature.

[110] Bartkova J., Hořejší Z., Koed K., Krämer A., Tort F., Zieger K., Guldberg P., Sehested M., Nesland J.M., Lukas C. (2005). DNA Damage Response as a Candidate Anti-Cancer Barrier in Early Human Tumorigenesis. Nature.

[111] Glück S., Guey B., Gulen M.F., Wolter K., Kang T.-W., Schmacke N.A., Bridgeman A., Rehwinkel J., Zender L., Ablasser A. (2017). Innate Immune Sensing of Cytosolic Chromatin Fragments through CGAS Promotes Senescence. Nat. Cell Biol..

[112] Peto J. (2001). Cancer Epidemiology in the Last Century and the next Decade. Nature.

[113] Ahmad A.S., Ormiston-Smith N., Sasieni P.D. (2015). Trends in the Lifetime Risk of Developing Cancer in Great Britain: Comparison of Risk for Those Born from 1930 to 1960. Br. J. Cancer.

[114] Amor-Guéret M. (2006). Bloom Syndrome, Genomic Instability and Cancer: The SOS-like Hypothesis. Cancer Lett..

[115] Kaur E., Agrawal R., Sengupta S. (2021). Functions of BLM Helicase in Cells: Is It Acting Like a Double-Edged Sword?. Front. Genet..

[116] Atsumi Y., Fujimori H., Fukuda H., Inase A., Shinohe K., Yoshioka Y., Shikanai M., Ichijima Y., Unno J., Mizutani S. (2011). Onset of Quiescence Following P53 Mediated Down-Regulation of H2AX in Normal Cells. PLoS ONE.

[117] Atsumi Y., Minakawa Y., Ono M., Dobashi S., Shinohe K., Shinohara A., Takeda S., Takagi M., Takamatsu N., Nakagama H. (2015). ATM and SIRT6/SNF2H Mediate Transient H2AX Stabilization When DSBs Form by Blocking HUWE1 to Allow Efficient ΓH2AX Foci Formation. Cell Rep..

[118] Minakawa Y., Atsumi Y., Shinohara A., Murakami Y., Yoshioka K. (2016). Gamma-Irradiated Quiescent Cells Repair Directly Induced Double-Strand Breaks but Accumulate Persistent Double-Strand Breaks during Subsequent DNA Replication. Genes Cells.

[119] Lukačišinová M., Novak S., Paixão T. (2017). Stress-Induced Mutagenesis: Stress Diversity Facilitates the Persistence of Mutator Genes. PLoS Comput. Biol..

[120] Fitzgerald D.M., Hastings P.J., Rosenberg S.M. (2017). Stress-Induced Mutagenesis: Implications in Cancer and Drug Resistance. Annu. Rev. Cancer Biol..

[121] Zhang J., Stevens M.F.G., Bradshaw T.D. (2012). Temozolomide: Mechanisms of Action, Repair and Resistance. Curr. Mol. Pharmacol..

[122] Yoshioka K., Yoshioka Y., Hsieh P. (2006). ATR Kinase Activation Mediated by MutSα and MutLα in Response to Cytotoxic O6-Methylguanine Adducts. Mol. Cell.

[123] Van Nifterik K.A., van den Berg J., Stalpers L.J.A., Lafleur M.V.M., Leenstra S., Slotman B.J., Hulsebos T.J.M., Sminia P. (2007). Differential Radiosensitizing Potential of Temozolomide in MGMT Promoter Methylated Glioblastoma Multiforme Cell Lines. Int. J. Radiat. Oncol. Biol. Phys..

[124] Ichijima Y., Yoshioka K., Yoshioka Y., Shinohe K., Fujimori H., Unno J., Takagi M., Goto H., Inagaki M., Mizutani S. (2010). DNA Lesions Induced by Replication Stress Trigger Mitotic Aberration and Tetraploidy Development. PLoS ONE.

